# A Possible Reason to Induce Acute Graft-vs.-Host Disease After Hematopoietic Stem Cell Transplantation: Lack of Sirtuin-1 in CD4^+^ T Cells

**DOI:** 10.3389/fimmu.2018.03078

**Published:** 2018-12-21

**Authors:** Ya-jing Xu, Fang-ping Chen, Yan Chen, Bin Fu, En-yi Liu, Lang Zou, Lin-xin Liu

**Affiliations:** Department of Hematology, Xiangya Hospital, Central South University, Changsha, China

**Keywords:** aGVHD, SIRT1, mTOR pathway, IL-1β signaling, Th17

## Abstract

Sirtuin 1 (SIRT1) is a critical suppressor of T cell immunity. However, whether SIRT1 is involved in the progression of acute graft-vs.-host disease (aGVHD) has still remained unclear. PI3K/Akt/mTOR pathway is a crucial element involved in the activation and functions of T cells. Over-activation of PI3K/Akt/mTOR signaling may be related to the occurrence of aGVHD. STAT3 activation requires phosphorylation and acetylation. A recent study showed that STAT3 hyperphosphorylation in CD4^+^ T cells may be a trigger of aGVHD. The role of the STAT3 acetylation in aGVHD pathogenesis is still unclear. The present study revealed that SIRT1 deficiency as a critical factor is involved in the excessive activation of mTOR pathway and upregulation of STAT3 acetylation and phosphorylation in CD4^+^ T cells from patients with aGVHD. Exorbitant activation of IL-1β signaling is the main reason for TAK1-dependent SIRT1 insufficiency. The findings of the present study might provide a new therapeutic target for treating aGVHD.

## Introduction

Allogeneic hematopoietic stem cell transplantation (allo-HSCT) is a lifesaving therapy for a variety of hematological disorders. However, acute graft-vs.-host disease (aGVHD) is a frequent and unpredictable severe inflammatory complication that seriously endangers patients' life. The process of aGVHD is associated with alloreactive donor T lymphocytes and their secretion of proinflammatory cytokines, resulting in inflammation in the gut, liver, and skin (1–4).

Sirtuin 1 (SIRT1) is a member of the sirtuin family of proteins that belongs to class III histone deacetylases (HDACs). It is highly expressed in heart, brain, skeletal muscle, and thymus (5). Recently, SIRT1 has emerged as a critical immune modulator by suppressing inflammation or regulating immune cell activation. In macrophages and dendritic cells (DCs), SIRT1 inhibits the production of proinflammatory cytokines (6). In myeloid-derived suppressor cells (MDSCs), SIRT1 directs the differentiation of MDSCs in antitumor immunity (7). In T cells, SIRT1 is a critical suppressor of T-cell immunity by suppressing the transcription factors, such as nuclear factor kappa light chain enhancer of activated B cells (NF-κB) and activator protein-1 (AP-1). Furthermore, SIRT1 may also negatively regulate T-cell activation via deacetylation in the promoter region to inhibit transcription of Bclaf1 (8–10). SIRT1-deficient mice showed elevated T cell activation and a lupus-like autoimmune phenotype (11, 12). However, whether SIRT1 can be involved in the progression of aGVHD has still remained unclear.

Activation of phosphatidylinositol 3-kinase/Akt/mammalian target of rapamycin (PI3K/Akt/mTOR) pathway in T cells promotes survival, modulates differentiation, and controls the acquisition of effector and memory phenotypes (13–16). Thus, this pathway is a crucial element involved in the activation and functions of T cells. Recent studies have indicated the role of mTOR activation in the initiation of inflammatory cascades. The inhibition of mTOR has been reported to improve the outcomes of various inflammatory and autoimmune diseases (17, 18). In particular, the inhibition of PI3K/Akt/mTOR pathway significantly improved the survival and decreased the GVHD development in mice (19). These findings strongly suggested the involvement of PI3K/Akt/mTOR in the progression of aGVHD.

Activation of signal transducer and activator of transcription 3 (STAT3) requires phosphorylation on tyrosine 705 (Tyr705) and serine 727 (Ser727) (20, 21). In addition, pSTAT3 on Tyr705 induces its dimerization, which is required for nuclear translocation and binding its target gene promoters, such as RORγt (22, 23). A recent study showed that pSTAT3 on Tyr705 increased significantly in CD4^+^ T cells among human recipients of allo-HSCT before the onset of Grade II–IV aGVHD (24). STAT3 acetylation on lysine 685 (Lys685) is another crucial post-translational modification for dimer stability and transcriptional activity. SIRT1-mediated deacetylation can inhibit STAT3 function, and deletion of SIRT1 can increase STAT3 acetylation and activity (25, 26). Several studies have confirmed the role of STAT3 acetylation in psoriasis (27), lymphoid malignancies (28), and hepatocellular carcinoma (29). However, the role of STAT3 acetylation in aGVHD pathogenesis is still elusive.

The present study found that SIRT1 deficiency is critically involved in the excessive activation of mTOR pathway and upregulation of STAT3 acetylation and phosphorylation in CD4^+^ T cells from patients with aGVHD. Exorbitant activation of IL-1β signaling is the main reason for TAK1-dependent SIRT1 insufficiency. The blockade of TAK1 could restore the expression of SIRT1. The findings of the present study might provide new therapeutic targets for treating aGVHD as well.

## Materials and Methods

### Patients

A total of 92 patients who underwent allo-HSCT (2014–2017) from HLA-identical sibling donors at the Key Laboratory of Hematopoietic Stem Cell Transplantation, Xiangya Hospital, Central South University, Changsha, China, were included in this study. The characteristics of these patients are shown in Table 1. This study was carried out in accordance with the recommendations of international ethical guidelines for biomedical research involving human subjects. The protocol was approved by the Human Ethics Committee of the Xiangya School of Medicine, Central South University. All subjects signed written informed consent in accordance with the Declaration of Helsinki. The adoption of conditioning regimen mainly depends on the type of disease and general status of patients. Acute myeloid leukemia and myelodysplastic syndrome: cytarabine [2 g/(m^2^. day), days −8 to −7], busulfan [3.2 mg/(kg. day), intravenously days −6 to −4)], cyclophosphamide [1.8 g/(m^2^. day), days −3 to −2], and semustine (250 mg/m^2^, day −2). Acute lymphocytic leukemia: total body irradiation (TBI) (5 Gy per day, days −5 to −4) and cyclophosphamide [1.8 g/(m^2^. day), days −3 to −2]. Chronic myeloid leukemia: busulfan [3.2 mg/(kg. day), intravenously days −8 to −5], cyclophosphamide [1.8 g/(m^2^. day), days −4 to −3], and semustine (250 mg/m^2^, day −2). Granulocyte colony-stimulating factor was used for mobilization of peripheral blood hematopoietic stem cells. The GVHD prophylaxis regimen was consisted of a combination of cyclosporine A (CsA) and short-term methotrexate (MTX). The dosage of CsA was 2.5 mg/(kg. day), intravenously from day −9. The dosage of MTX was 15 mg/m^2^ administered intravenously on day +1 and 10 mg/m^2^ on days +3 and +6.

**Table 1:** Clinical characteristics of patients.

	**No GVHD**	**aGVHD**
Number	46	46
Median age	33	37
Sex (female/male)	20/26	19/27
**DIAGNOSIS**		
ALL	13	15
AML	21	23
MDS	8	6
CML	4	2
**ACUTE GVHD GRADE**		
1		3
2		30
3		12
4		1
Days to aGVHD onset, median (range)		52 (range: 23–89)

Assessment of aGVHD was carried out based on clinical symptoms in accordance with commonly accepted criteria (30, 31). Organ lesions of skin, liver, and gastrointestinal tract were staged 1 through 4 for aGVHD. The patients were also assigned a grade of aGVHD (1 through 4) based on overall severity. aGVHD <grade 2 was continuously treated according to the prophylaxis regimen. aGVHD ≥ grade 2 was treated with methylprednisolone [2 mg/(kg. day)], MTX, and anti-CD25 monoclonal antibody were given to patients intolerant of or unresponsive to methylprednisolone.

We collected samples of patients at the onset of aGVHD (*n* = 46) and samples of patients without aGVHD (*n* = 46) at the same time points. Peripheral blood samples were collected as soon as aGVHD was diagnosed before starting the therapy, and then CD4^+^ T cells were isolated. The isolated CD4^+^ T cells were used for culture or cryopreservation in −70°C sample library.

### Isolation and Culturing of CD4^+^ T Cells

CD4^+^ T cells were purified from 60 mL of venous peripheral blood from patients with aGVHD using human CD4 beads, according to the manufacturer's protocol (Miltenyi Biotec, Bergisch Gladbach, Germany). The isolated CD4^+^ T cells were cultured in human T cell culture medium (Lonza, Basel, Switzerland), and supplemented with 10% fetal bovine serum (FBS) and 1% penicillin/streptomycin.

For CD4^+^ T cell stimulation *in vitro*, the cells were plated at a density of 1–5 × 10^6^/well on 24-well plates containing 2.5 mg/mL plate-bound anti-CD3 with 1 mg/mL soluble anti-CD28 monoclonal antibodies (eBioscience, San Diego, CA, USA).

### Cell Activity Assay and Flow Cytometry

After stimulation using anti-CD3/anti-CD28 antibodies, CD4^+^ T cells were inoculated on a 96-well plate (1 × 10^4^/well) with/without 5 μM SRT1720. The plate was incubated at 37°C in presence of 5% CO_2_. Then, 10 μL of Cell Counting Kit-8 (CCK-8) was added to each well of the plate. The plate was incubated for 1 h in the incubator. The absorbance was measured at 450 nm using a microplate reader.

CD4^+^ T cells were also examined for the expression of cell surface CD25 and intracellular interferon (IFN)-γ using fluorophore-conjugated mAbs (eBioscience, San Diego, CA, USA). Isotype-matched immunoglobulin Gs were used as controls. Besides, the BD FACSCanto II system (BD Biosciences, CA, USA) was used for data acquisition, and data were analyzed using FlowJo software.

### Cell Proliferation Assay

CD4^+^ T cells were separated from 40 ml heparinized blood of 3 healthy donors by immunomagnetic beads and labeled with CFSE (eBioscience). The labeled CD4^+^ T cells were activated with anti-CD3/anti-CD28 antibodies, and then IL-2 was added to expand the CD4^+^ T cells. The activated CD4^+^ T cells were inoculated on a 6-well plate (5 × 10^5^/well) with/without 5 μM SRT1720. The plate was incubated at 37°C in presence of 5% CO_2_ for 3 days. The proliferation of CD4^+^ T cells were detected by flow cytometry.

### Mixed Lymphocyte Reaction (MLR)

We followed the method of Stella et al. (32): Peripheral blood mononuclear cells (PBMCs) were separated from 20 ml heparinized blood of 3 healthy donors by density gradient centrifugation. PBMCs were resuspended at a concentration of 2 × 10^6^ cells/mL in complete medium (RPMI-1640 containing 10% fetal bovine serum). RPMI 1788 cells (human B lymphocyte cell line) were treated with 30 μg/mL of mitomycin C for 20 min at 37°C, washed four times with medium and finally suspended in complete medium to a density of 1 × 10^6^ cells/mL. One hundred microliters of each cell suspension were mixed with/without 5 μM SRT1720 in 96-well microtiter plates as experimental group, 100 μL of PBMCs suspension was mixed with complete medium in 96-well microtiter plates as negative control group. The mixed cells were cultured for 6 days at 37°C in a humidified atmosphere of 5% CO_2_ and 95% air. DNA synthesis was assayed by the addition of 1 μCi 3H-TdR per well during the last 18 h of culture. Thereafter, the cells were harvested on glass filter paper and the counts per minute determined in a liquid scintillation counter. Stimulating index (SI) = CPM of experimental group − CPM of blank/CPM of negative control group − CPM of blank.

### Quantitative Real-Time Polymerase Chain Reaction (qPCR)

Total RNA was isolated from CD4^+^ T cells using TRIzol Reagent (Invitrogen, CA, USA). RNA was reverse transcribed into cDNA using random primer and SuperScript II reverse transcriptase (Invitrogen, CA, USA), according to the manufacturer's protocol. The qPCR was performed in triplicates using SYBR Green Master Mix (ABI Prism 7500, CA, USA). Human glyceraldehyde-3-phosphate dehydrogenase (GAPDH) gene was used as an endogenous control for sample normalization. The fold change was calculated using the following formula 2^−ΔΔCt^: ΔΔCt = (Ct_target gene_ – Ct_internal control_)_sample_ – (Ct_target gene_ – Ct_internal control_)_control_. The primers are listed in Table 2.

**Table 2:** Primer sequences for real-time qPCR.

	**Forward primer**	**Reverse primer**
SIRT1	TACCGAGATAACCTTCTG	TCCAGTCACTAGAGCTTG
RORγt	GCTGGTTAGGATGTGCCG	GGATGCTTTGGCGATGA
IL17	CAATCCCACGAAATCCAGGATG	GGTGGAGATTCCAAGGTGAGG
IL17f	TGCTCAAGGAAAGGAAGACA	ATGGTGGATGACAGGGGTG
IL-22	AGCTTGGCTGATAACAACACA	AAGGGCACCACCTCCTG
GAPDH	AAGAGCTACGAGCTGCCTGAC	ATGGCCCAGCGGATGAG

### Western Blot Analysis

CD4^+^ T cells were lysed in protein lysis buffer containing proteinase inhibitor (Therm-Pierce). Lysates were centrifuged at 14,000 g and 4°C for 15 min, and protein concentration was determined using the Bradford protein assay (Bio-Rad, CA, USA). Proteins were loaded and separated by electrophoresing with an 8% sodium dodecyl sulfate–polyacrylamide gel electrophoresis (SDS-PAGE), and then electrophoretically transferred onto polyvinylidene difluoride membrane (PVDF; Bio-Rad, CA, USA). The membranes were blocked with 5% bovine serum albumin (BSA) for 1 h at room temperature, washed twice with Tris-buffered saline with Tween 20 (TBST), and then separately incubated with primary antibodies overnight at 4°C. The antibodies included anti-SIRT1, anti-Akt, anti-phospho-Akt (Ser473 and Thr308), anti-mTOR, anti-phospho-mTOR (Ser2448), anti-4E-BP1, anti-phospho-4E-BP1 (Thr37/46), anti-STAT3, anti-phospho-STAT3 (Tyr705), anti-acetyl-STAT3 (Lys685), anti-IRAK4, anti-phospho-IRAK4 (Thr345/Ser346), anti-TAK1, anti-phospho-TAK1 (Thr187 and Ser412), and anti-GAPDH. All antibodies were obtained from Cell Signaling Technology (MA, USA). The membranes were washed with TBST and incubated with horseradish peroxidase–conjugated secondary antibodies for 2 h at room temperature. The band intensity was quantified using Quantity One software (Bio-Rad, CA, USA).

### Enzyme-Linked Immunosorbent Assay of Interleukin−1β Protein Level

The concentrations of serum interleukin-1β (IL-1β) was measured using the Human IL-1β enzyme-linked immunosorbent assay (ELISA) kit (Abcam, Cambridge, UK), according to the manufacturer's instruction. The absorbance at 450 nm was recorded using a microplate reader.

### Statistical Analysis

The paired *t*-test was used for comparing matched datasets. The unpaired Student *t*-test or Mann–Whitney test was used for comparing independent datasets based on Gaussian distribution. Analysis of variance (ANOVA) was used for making comparison between groups. All analyses were performed using SPSS 16.0 software (SPSS Inc., IL, USA). The significance was set at *P* ≤ 0.05.

## Results

### Patients

Among the 92 patients with HSCT, 46 cases presented with aGVHD and 46 cases didn't have aGVHD. Of the 46 patients who developed aGVHD, 3 (6.5%) had grade 1, 30 (65.2%) had grade 2, 12 (26.1%) had grade 3, and 1 (2.2%) had grade 4. The median day of onset of aGVHD was 52 (range: 23–89). Furthermore, 43 episodes of grades 2-4 aGVHD were treated with methylprednisolone, and 29 (67.4%) episodes were successfully treated, whereas 14 episodes that lacked adequate response to the primary treatment were treated with intravenous MTX (10 mg per day, 1–2 times per week) and anti-CD25 monoclonal antibody. All patients survived until the 100th day.

### SIRT1 Deficiency Enhanced Activation of CD4^+^ T Cells in Patients With aGVHD

**Figure 1:**
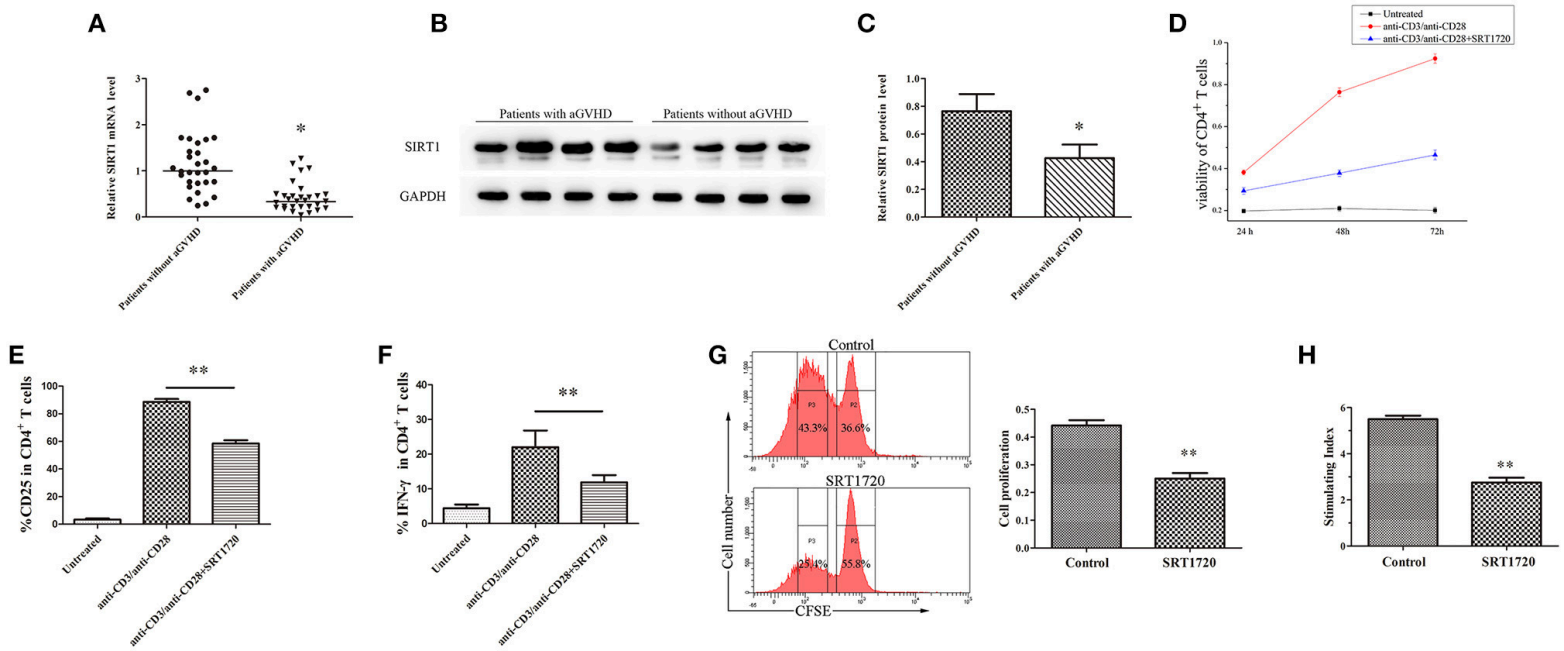
SIRT1 deficiency enhanced CD4^+^ T cell activation in patients with aGVHD. **(A)** Relative mRNA level of SIRT1 in CD4^+^ T cells from patients with aGVHD (*n* = 30) and patients without aGVHD (*n* = 30) normalized to GAPDH. **(B,C) (B)** Representative Western blotting result for SIRT1 protein expression in CD4^+^ T cells from patients with aGVHD (*n* = 10) and patients without aGVHD (*n* = 10). **(C)** Quantitative analysis of the band intensities for SIRT1 protein level normalized by GAPDH. **(D)** Determination of viability of CD4^+^ T cells unstimulated or stimulated, treated or not with SRT1720. **(E,F)** Percentage of CD25^+^ and IFN-γ^+^ cells among CD4^+^ T cells unstimulated or stimulated, treated or not with the SRT1720. **(G)** The CFSE labeled CD4^+^ T cells were activated with anti-CD3/anti-CD28 antibodies and IL-2, and treated with/without SRT1720. The proliferation of CD4^+^ T cells were detected by flow cytometry. **(H)** PBMCs and RPMI 1788 cells were mixed culture with/without SRT1720. The 3H-TdR incorporation was used to detect PBMCs proliferation. Data are presented as the mean ± standard deviation (SD) of the same experiments performed in three times. *p < 0.05, **p < 0.01.

The mRNA levels of SIRT1 were measured in CD4^+^ T cells from patients with aGVHD and patients without aGVHD. The results obtained from qPCR showed that the expression of SIRT1 was significantly downregulated in patients with aGVHD compared with patients without aGVHD (Figure 1A). Moreover, Western blot analysis confirmed the decrease of SIRT1 in CD4^+^ T cells from patients with aGVHD (Figures 1B,C).

CD4^+^ T cells from normal human donors who had plate-bound anti-CD3/anti-CD28 antibodies were stimulated and cultured for 72 h with/without 5 μM SRT1720 (33), a classical activator of SIRT1, to test the influence of SIRT1 on CD4^+^ T-cell activation. The CCK-8 kit was used to monitor the viability of CD4^+^ T cells. Cell surface expression of CD25 and intracellular expression of IFN-γ were analyzed by flow cytometry. Following TCR (T cell receptor) stimulation, SRT1720 significantly suppressed the viability (Figure 1D), and reduced the percentage of CD25 and IFN-γ (Figures 1E,F) in CD4^+^ T cells. Additional, we detected the effect of activated SIRT1 on the proliferation of CD4^+^ T cells by cell proliferation assay. The result showed that SRT1720 significantly inhibited the proliferation of anti-CD3/anti-CD28 antibodies and IL-2 stimulated CD4^+^ T cells (Figure 1G). In confirmation of the suppressive and regulatory role of SIRT1 in the pathology of aGVHD, we performed a mixed lymphocyte reaction. As showed in Figure 1H, SRT1720 remarkably restrained the activation effect of stimulating cells (RPMI 1788 cells) on lymphocytes. Taken together, the expression of SIRT1 was substantially downregulated in aGVHD CD4^+^ T cells. Besides, the restored expression of SIRT1 could suppress CD4^+^ T cell excessive activation.

### IL-1β Signaling Pathway Downregulated the Expression of SRIT1 in CD4^+^ T Cells From Patients With aGVHD

**Figure 2:**
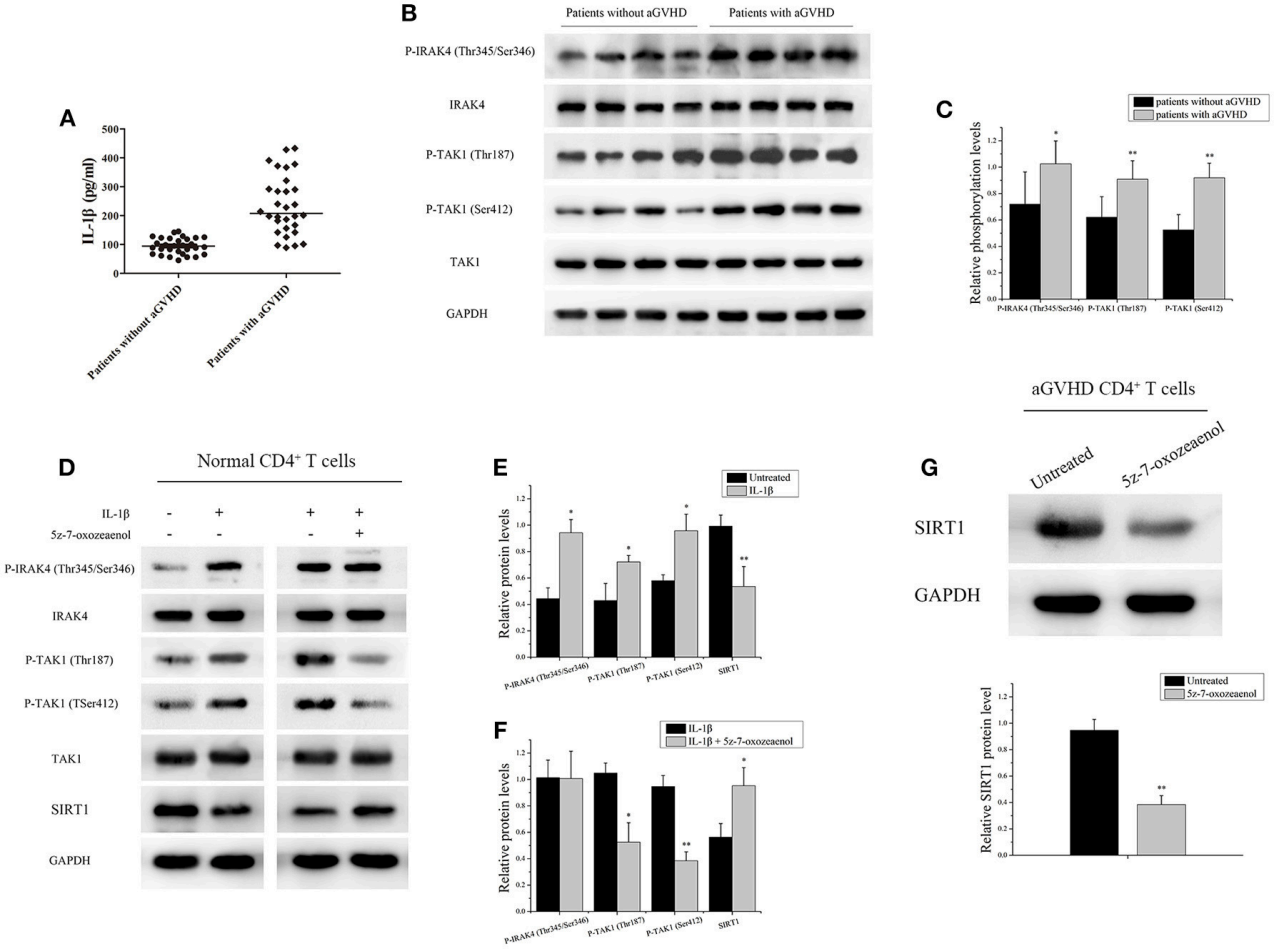
IL-1β signaling pathway downregulated the expression of SRIT1 in CD4^+^ T cells from patients with aGVHD. **(A)** The expression of IL-1β was investigated in serum from patients with (*n* = 30)/without (*n* = 30) aGVHD using ELISA. **(B)** IRAK4 and TAK1 phosphorylation levels in CD4^+^ T cells from patients with aGVHD (*n* = 10) and patients without aGVHD (*n* = 10) were detected by Western blotting. **(C)** Quantitative analysis of the band intensities for IRAK4 and TAK1 phosphorylation levels normalized by total protein levels. **(D–F)** Representative western blotting results **(D)** and quantitative analysis **(E,F)** of the band intensities for IRAK4 and TAK1 phosphorylation levels in normal CD4^+^ T cells treated or not with IL-1β/5z-7-oxozeaenol. **(G)** Representative western blotting results (top) and quantitative analysis (bottom) of the band intensities for SIRT1 protein level in CD4^+^ T cells from patients with aGVHD treated or not with 5z-7-oxozeaenol. Data are presented as the mean ± SD of the same experiments performed in three times. *p < 0.05, **p < 0.01.

IL-1β, an acute-phase proinflammatory cytokine, has been implicated in the pathophysiology of several autoimmune diseases. Receptor antagonists of IL-1β can effectively alleviate GVHD (34, 35). Thus, the expression of IL-1β was investigated in serum from patients with/without aGVHD using ELISA. The IL-1β level was found to be significantly increased in serum of patients with aGVHD compared with patients without aGVHD (Figure 2A). Moreover, the phosphorylation of IRAK4 and TAK1, which are critical intermediates of IL-1β signaling pathway, was assessed. IRAK4 phosphorylation at Thr345/Ser346 and TAK1 phosphorylation at Thr187 and Ser412 were substantially upregulated in patients with aGVHD compared with patients without aGVHD (Figures 2B,C).

CD4^+^ T cells from healthy donors with IL-1β were stimulated to ascertain the role of IL-1β signaling pathway in regulating the expression of SRIT1 in CD4^+^ T cells. As expected, the expression of SIRT1 was markedly reduced, whereas phosphorylation of IRAK4 and TAK1 was increased in the IL-1β-stimulated group compared with control (Figures 2D,E). Then, CD4^+^ T cells from healthy donors were treated with/without 5z-7-oxozeaenol, a potent TAK1 inhibitor, for 24 h following IL-1β stimulation. 5z-7-oxozeaenol significantly increased the expression of SIRT1 in IL-1β-stimulated CD4^+^ T cells (Figures 2D,F). Also, CD4^+^ T cells from patients with aGVHD were treated with 5z-7-oxozeaenol. Similarly, the expression of SIRT1 was reversed in the 5z-7-oxozeaenol-treated group (Figure 2G). Taken together, these data indicated that the excessive activation of IL-1β signaling pathway was one of the main reasons for SIRT1 deficiency in CD4^+^ T cells from patients with aGVHD. The blockade of TAK1 could restore the expression of SIRT1 as well.

### SIRT1 Targeting of mTOR Pathway to Inhibit CD4^+^ T Cell Activation

**Figure 3:**
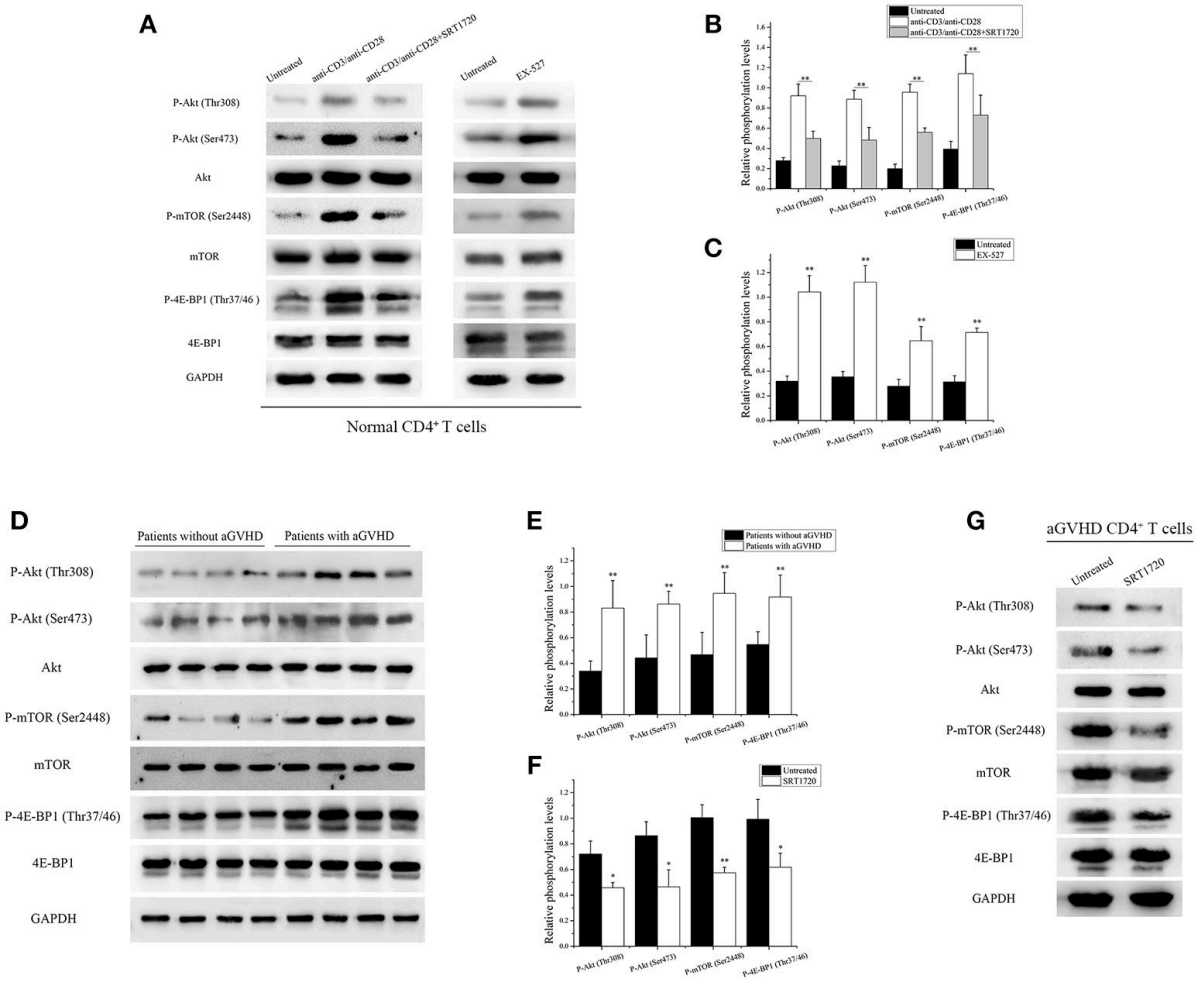
SIRT1 targeting of mTOR pathway to inhibit CD4^+^ T cell activation. **(A)** (left) Representative western blotting result for relative phosphorylation of Akt, mTOR, 4E-BP1 in CD4^+^ T cells unstimulated or stimulated, treated or not with the SRT1720. (right) Representative western blotting result for relative phosphorylation of Akt, mTOR, 4E-BP1 in CD4^+^ T cells treated or not with EX-527. **(B,C)** Quantitative analysis of the band intensities for phosphorylation levels normalized by total protein levels. **(D)** Representative western blotting result for relative phosphorylation of Akt, mTOR, 4E-BP1 in CD4^+^ T cells from patients with aGVHD (*n* = 10) and patients without aGVHD (*n* = 10). **(E,F)** Quantitative analysis of the band intensities for phosphorylation levels normalized by total protein levels. **(G)** Representative western blotting result for relative phosphorylation of Akt, mTOR, 4E-BP1 in CD4^+^ T cells from patients with aGVHD treated or not with SRT1720. Data are presented as the mean ± SD of the same experiments performed in three aGVHD patients. *p < 0.05, **p < 0.01.

PI3K/Akt/mTOR is a key signaling pathway involved in activation and function of T cells (13). CD4^+^ T cells from normal human donors were treated with/without SRT1720 for 6 h following TCR stimulation to investigate whether SIRT1 could inhibit the mTOR pathway mediating CD4^+^ T cell inaction. After treatment with SRT1720, a decrease in relative Akt phosphorylation at Thr308 and Ser473, relative mTOR phosphorylation at Ser2448, and relative 4E-BP1 phosphorylation at Thr37/46 were observed compared with TCR-stimulated CD4^+^ T cells without SRT1720 treatment (Figures 3A,B). In contrast, the inhibition of SIRT1 in CD4^+^ T cells from normal human donors using EX-527, a classical SIRT1 inhibitor, was observed. The phosphorylation of Akt, mTOR, and 4E-BP1 increased as well (Figures 3A,C).

Based on the aforementioned results, the phosphorylation of Akt, mTOR, and 4E-BP1 in CD4^+^ T cells from patients with aGVHD and patients without aGVHD was examined. The study further validated that the phosphorylation of Akt, mTOR, and 4E-BP1 substantially increased in CD4^+^ T cells from patients with aGVHD compared with patients without aGVHD (Figures 3D,E). After treatment with SRT1720, a decrease in Akt, mTOR, and 4E-BP1 phosphorylation was observed in aGVHD CD4^+^ T cells compared with control (Figures 3F,G). These data indicated that SIRT1 deficiency was a critical factor involved in the excessive activation of mTOR pathway in aGVHD CD4^+^ T cells. In addition, the activation of SIRT1 could inhibit the mTOR pathway.

### SIRT1 Deficiency in aGVHD CD4^+^ T Cells Promoted Th17-Related Gene Expression

**Figure 4:**
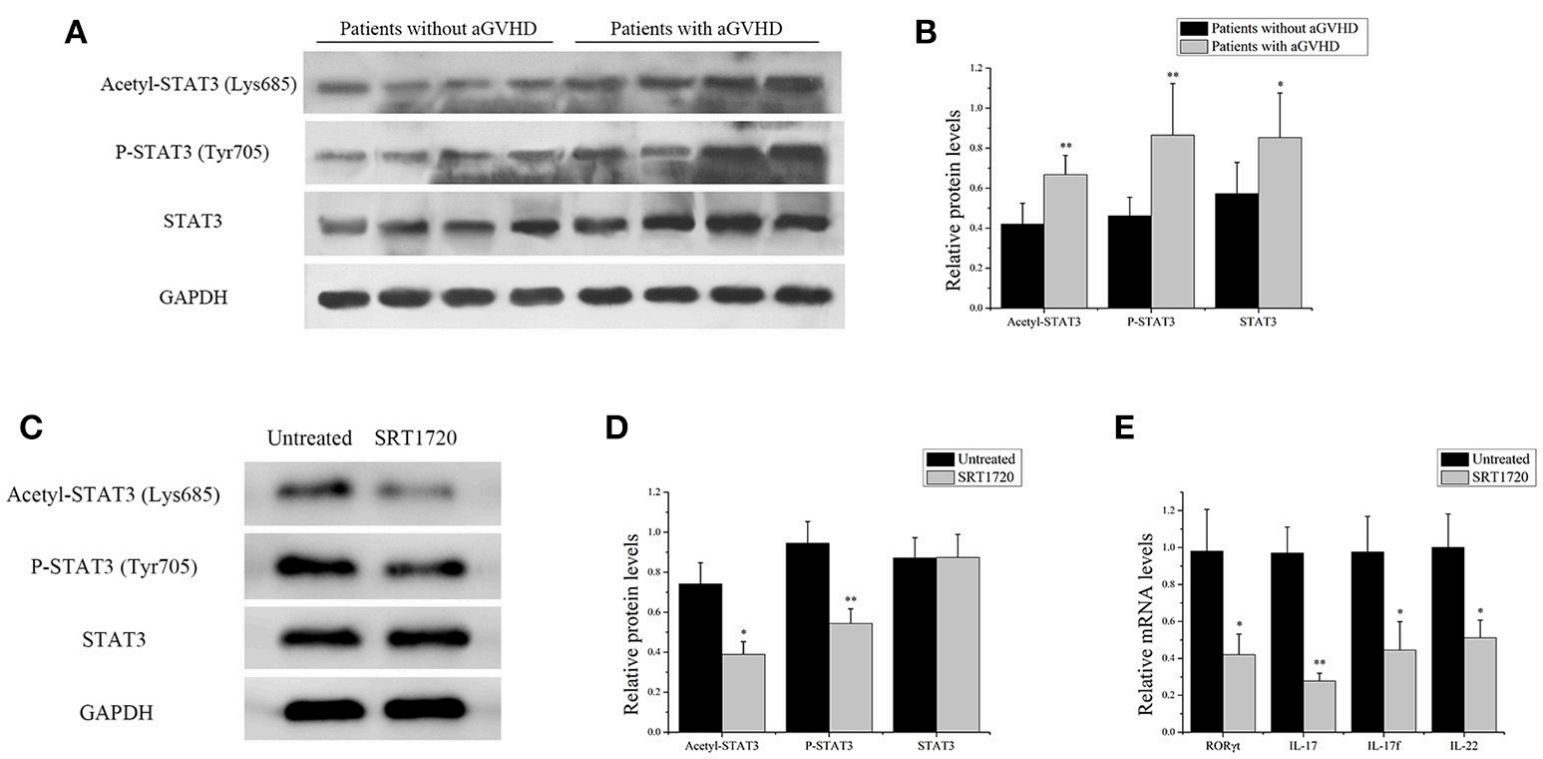
SIRT1 deficiency in CD4^+^ T cells from patients with aGVHD promoted Th17-related gene expression. **(A)** acetyl-STAT3 (Lys685), P-STAT3 (Tyr705), and STAT3 total protein levels in CD4^+^ T cells from patients with aGVHD (*n* = 10) and patients without aGVHD (*n* = 10) were detected by Western blotting. **(B)** Quantitative analysis of the band intensities for acetyl-STAT3 (Lys685) and P-STAT3 (Tyr705) normalized by STAT3 total protein levels, STAT3 total protein levels normalized by GAPDH. **(C,D)** Representative western blotting results **(C)** and quantitative analysis **(D)** of the band intensities for acetyl-STAT3 and P-STAT3 in CD4^+^ T cells from patients with aGVHD treated or not with the SRT1720. **(E)** mRNA levels of RORγt, IL17, IL17f, IL-22 in CD4^+^ T cells from patients with aGVHD treated or not with SRT1720. Data are presented as the mean ± SD of the same experiments performed in three aGVHD patients. *p < 0.05, **p < 0.01.

The acetylation of STAT3 on Lys685 and phosphorylation of STAT3 on Tyr705 were measured in CD4^+^ T cells from patients with aGVHD and without aGVHD. The results showed that the expression of total STAT3 was higher in CD4^+^ T cells of patients with aGVHD compared with those without aGVHD. Additionally, STAT3 acetylation on Lys685 and phosphorylation on Tyr705 were significantly upregulated in patients with aGVHD compared with patients without aGVHD (Figures 4A,B).

CD4^+^ T cells from patients with aGVHD were treated with SIRT1 agonist to further demonstrate the impact of SIRT1 on STAT3 acetylation and phosphorylation in CD4^+^ T cells from patients with aGVHD. The Western blot analysis result showed that the STAT3 acetylation on Lys685 and phosphorylation on Tyr705 significantly decreased in the SRT1720 treatment group (Figures 4C,D). The expression of RORγt, IL-17, IL-17f, and IL-22 after SRT1720 treatment in CD4^+^ T cells from patients with aGVHD was assessed using qPCR to determine the effect of SIRT1 agonists on the expression of Th17-related gene. The expression of RORγt, IL-17, IL-17f, and IL-22 was specifically reduced in the SRT1720 treatment group (Figure 4E). Overall, these findings suggested that SIRT1 deficiency in CD4^+^ T cells from patients with aGVHD might spur STAT3 acetylation and phosphorylation. Moreover, the activation of SIRT1 could inhibit STAT3 acetylation and phosphorylation and Th17-related gene expression.

## Discussion

Recent studies revealed that SIRT1 was required for peripheral CD4^+^ T-cell tolerance. SIRT1-deficient T cells showed dramatically the increased proliferation and produced more IL-2 when stimulated with anti-CD3 and anti-CD28 antibodies *in vitro* (5, 8). Breakdown of CD4^+^ T-cell tolerance is crucial in the development of autoimmune diseases (36, 37). SIRT1-null mice not only develop systemic lupus erythematous-like symptoms spontaneously, but also are more susceptible to induced experimental autoimmune encephalomyelitis (EAE) (11). Pharmacological modulation of SIRT1 activity has been used as a therapeutic strategy in autoimmune disease settings. Resveratrol, a SIRT1 activator, serves as an anti-inflammatory compound and can alleviate various inflammatory diseases, such as rheumatoid arthritis and type I diabetes (38–40). Treatment of nonobese diabetic mice with resveratrol not only reduced proinflammatory cytokine production and lymphocyte activation, but also suppressed lymphocyte infiltration by decreasing the expression of CCR6 (39). The present study showed that the expression of SIRT1 was significantly downregulated in CD4^+^ T cells from patients with aGVHD compared with patients without aGVHD. Treatment of CD4^+^ T cells from patients with aGVHD with SIRT1 agonist could significantly suppress cell activation. These results suggested that the lack of SIRT1 in CD4^+^ T cells led to induction of aGVHD.

It is well-known that mTOR exists as a gene, however, it forms two distinct protein complexes: mTOR complex 1 (mTORC1) and mTORC2. mTORC1 has a critical role in regulating diverse cellular processes, including transcription, ribosome biogenesis, and protein synthesis, through its targets RPS6 and 4E-BP1 (13, 41). Stimulation of T-cell receptor, costimulatory molecules (CD28), and cytokine receptors leads to the activation of mTORC1 (13). The activation of PI3K, through the production of PIP3, activates PDK1, which in turn activates Akt as well. Activated Akt is crucial for the activation of mTORC1 (42, 43). Rapamycin, a specific inhibitor of mTOR, prevented naïve T-cell activation and induced tolerance of alloreactive T cells, significantly improving the survival, as well as decreasing the GVHD development in mice (19, 44). This study found that the phosphorylation of Akt, mTOR, and 4E-BP1 was substantially increased in CD4^+^ T cells from patients with aGVHD. Thus, activation of PI3K/Akt/mTOR signaling pathway may be vital in GVHD development. SIRT1, as a negative regulator, has been implicated in the modulation of PI3K/Akt pathway by deacetylation of the tumor suppressor PTEN (45) and down-regulation of both Akt and phosphorylation levels to inhibit the PI3K/Akt pathway in glioblastoma cells (46). In the present study, a decrease in Akt, mTOR, and 4E-BP1 phosphorylation was observed in CD4^+^ T cells from a patient with aGVHD after treatment with SIRT1 activator. These data indicated that SIRT1 deficiency was a critical factor involved in excessive activation of PI3K/Akt/mTOR pathway in CD4^+^ T cells from patients with aGVHD.

STAT3, as a vital regulator of Th17 differentiation and function, was involved in the pathogenesis of aGVHD. Numerous *in vitro* studies have shown that STAT3 can activate downstream of receptors for several proinflammatory cytokines, including IL-6, IL-21, and IL-23, leading to the upregulation of the expression of RORγt, RORα, IL-21, IL-23R, and IL-17 along with the development and stabilization of Th17 cells (47–50). Laurence et al. demonstrated that murine recipients of allo-HSCT with T lymphocytes lacking STAT3 exhibited conspicuously persistent survival compared with mice that received allogeneic transplants with control T lymphocytes (51). The present study revealed that STAT3 acetylation on Lys685 and phosphorylation on Tyr705 were significantly upregulated in patients with aGVHD compared with patients without aGVHD. Besides, SIRT1 agonists could significantly decrease STAT3 acetylation on Lys685 and phosphorylation on Tyr705. The expression of Th17-related gene, RORγt, IL-17, IL-17f, and IL-22 was specifically reduced after treatment with SIRT1 agonists. Hence, SIRT1 could be a key factor to inhibit STAT3 acetylation in CD4^+^ T cells, and the lack of SIRT1 could cause STAT3 hyperacetylation in CD4^+^ T cells from patients with aGVHD, which might also influence STAT3 phosphorylation partially.

The proinflammatory cytokines (IL-1β, TNF-α, IFN-γ, and IL-6) are crucial in the progression of GVHD (52, 53). Blockade of IL-1β has been shown to reduce aGVHD-related mortality in mice (52, 54). TAK1 is a member of the MAPKKK family, and is activated by various cytokines, including the family of TGF-β ligands (55). Increasing evidence has shown the involvement of TAK1 in the IL-1β signaling pathway. Following exposure of cells to IL-1β, endogenous TAK1 is recruited to the TRAF6 complex and activated. Activated TAK1 then stimulates both JNK and NF-kB activation (56, 57). This study demonstrated that the serum IL-1β level significantly increased, and the phosphorylation of downstream signaling molecules IRAK4 and TAK1 in CD4^+^ T cells was substantially upregulated in patients with aGVHD. Furthermore, the expression of SIRT1 was markedly reduced after stimulation with IL-1β in CD4^+^ T cells from healthy donors. TAK1 inhibitor could revert the expression of SIRT1 in IL-1β stimulated CD4^+^ T cells. These data revealed that the excessive activation of IL-1β signaling pathway was one of the main reasons for SIRT1 deficiency in CD4^+^ T cells from patients with aGVHD.

## Conclusions

**Figure 5:**
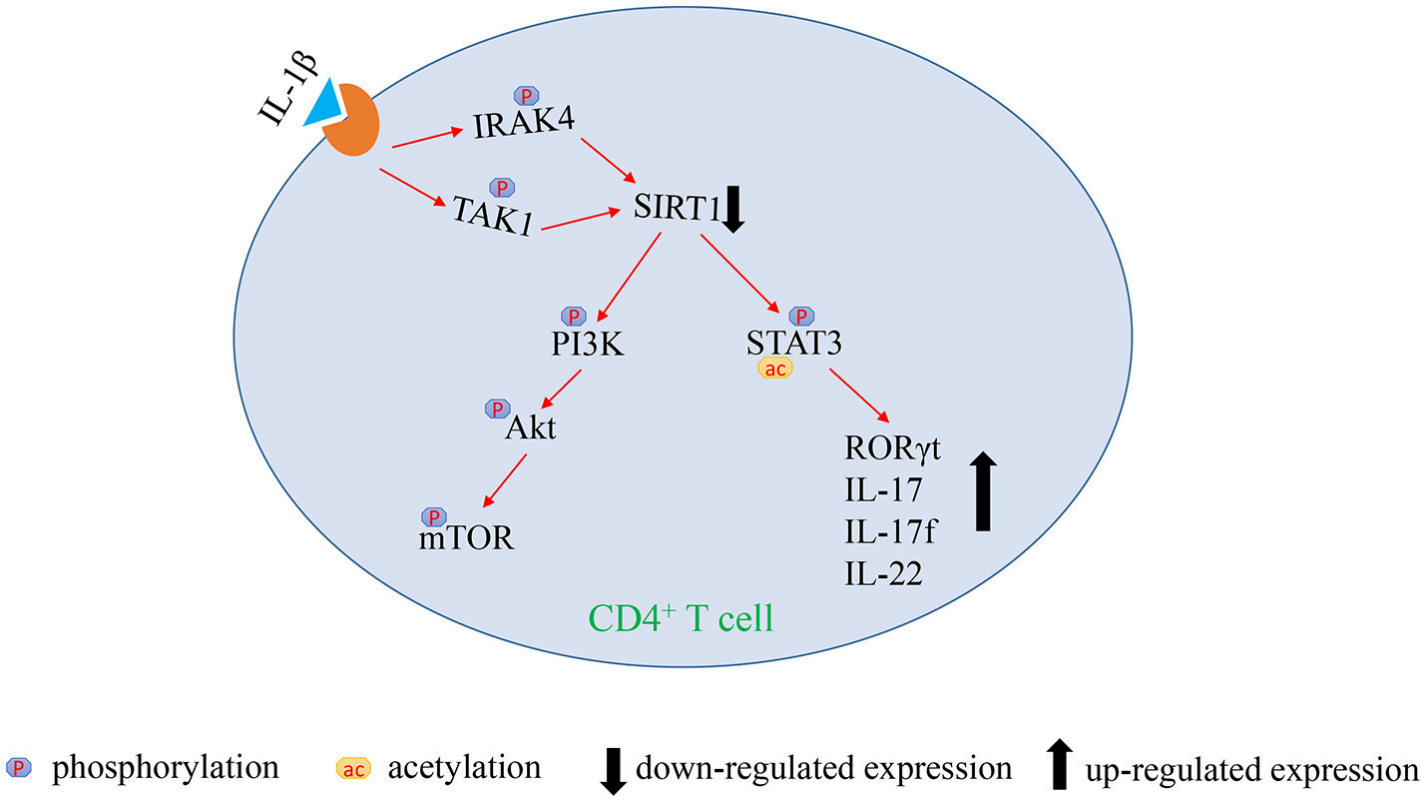
Figure illustrating our hypothesis of signaling regulation in aGVHD.

In summary, this study indicates that the over-activated IL-1β signaling pathway mediates SIRT1 deficiency in CD4^+^ T cells. The lack of SIRT1 leads to excessive activation of PI3K/Akt/mTOR pathway and STAT3 hyperacetylation and hyperphosphorylation, which promotes the excessive activation and the release of proinflammatory cytokine in CD4^+^ T cells, thereby inducing aGVHD (Figure 5). Our study highlighted an innovative molecular mechanism of the pathological process of aGVHD, and provided a new target for clinical treatment as well.

## Author Contributions

YX and FC designed experiments. YC, BF, and EL carried out experiments. YX and LZ analyzed experimental results. YX and LL wrote the manuscript.

### Conflict of Interest Statement

The authors declare that the research was conducted in the absence of any commercial or financial relationships that could be construed as a potential conflict of interest.
